# Gendered eating: Can gender role orientations explain gender differences in healthy eating?

**DOI:** 10.1177/13591053251388269

**Published:** 2025-11-20

**Authors:** Agnes Effert, Katharina Naomi Eichin, Gudrun Sproesser

**Affiliations:** 1 Johannes Kepler University Linz, Austria

**Keywords:** healthy eating, Health Action Process Approach, gender role orientations, femininity, masculinity

## Abstract

Although differences in healthy eating between women and men have been documented, with women generally adhering to healthier diets, the underlying mechanisms are not fully understood. Therefore, the current study investigated whether gender role orientations (i.e. an individual’s identification with certain characteristics that are traditionally associated with femininity and masculinity) mediate the relation between gender and healthy eating as well as with its antecedents according to the Health Action Process Approach (i.e. risk perception, outcome expectancies, self-efficacy, and intention). In total, 825 participants engaged in a pre-registered, cross-sectional online survey. Both feminine and masculine gender role orientations were positively associated with healthy eating and its antecedents – except masculinity for risk perception – and mediated their relation with gender accordingly. The results challenge the previous assumption, that masculinity is detrimental to healthy eating and highlight the potential for leveraging both – femininity- and masculinity-related aspects in designing gender-sensitive health interventions.

## Introduction

Poor nutrition is a major global health threat and is linked to higher rates of mortality than tobacco use, alcohol consumption, and unsafe sexual practices combined (Willett et al., 2019). Obesity and non-communicable diseases result from a complex interplay of factors; among these, unhealthy diets constitute a major modifiable risk factor (World Health Organization, 2020) and substantially reduce healthy years of life (World Health Organization Regional Office for Europe, 2004). Given these severe consequences, a better understanding of the factors associated with eating behavior is needed to promote public health.

Gender and sex differences^1^ in healthy eating have been reported across several studies, with women generally adhering to healthier diets than men (Arganini et al., 2012; Hiza et al., 2013; Imamura et al., 2015). For example, women report consuming more fruits and vegetables (Beardsworth et al., 2002; Prättälä et al., 2007; Wardle et al., 2004), but less red and processed meat (Bärebring et al., 2020; Beardsworth et al., 2002; Clonan et al., 2016; Prättälä et al., 2007), and salt (Heller et al., 2023; Wardle et al., 2004) than men. However, categorizing individuals as female or male is increasingly criticized as overly simplistic and insufficient to capture the complexity of gendered behaviors (Hyde et al., 2019). Consequently, scholars recently called for more attention to the within-gender heterogeneity in health behaviors (Greaves and Ritz, 2022; Rosenfeld and Tomiyama, 2021) and there is a recognized need to not only mention gender differences but also to understand the underlying mechanisms (Wardle et al., 2004).

One potential mechanism that may help explain these differences between women and men in eating behavior is the concept of gender role orientations – that is, an individual’s identification with characteristics stereotypically associated with femininity (e.g. caregiving, tenderness, and family orientation) and masculinity (e.g. risk-taking, pragmatism, and adventurousness) (Bem, 1974). Femininity and masculinity are conceptualized as two independent dimensions, and individuals can identify with characteristics of one dimension, both, or neither, irrespective of their sex and gender (Bem, 1974). While women score higher on feminine characteristics than men and vice versa, considerable within-gender variability exists (Bem, 1974; Calvo-Salguero et al., 2008; Spence and Buckner, 2000). The concept of psychological androgyny, as originally proposed by Bem (1974), refers to individuals who endorse both feminine and masculine traits to a high degree. Androgyny has been proposed to foster greater behavioral flexibility and more psychological well-being (Bem, 1974). Nevertheless, the current study focuses specifically on femininity and masculinity as separate dimensions to disentangle their potentially distinct associations with eating behavior.

Gender role orientations have been associated with meat consumption (Stanley et al., 2023), disordered eating (Hepp et al., 2005), and adherence to the Mediterranean diet (González-Pascual et al., 2024). However, research on their relation with healthy eating more broadly remains scarce (Fleming and Agnew-Brune, 2015). To address this gap, the current study investigates whether gender role orientations can explain the relationship between gender and healthy eating.

Differences in healthy eating between women and men might also be explained by differences in the social-cognitive antecedents of health behavior, postulated in health behavior models. For instance, the Health Action Process Approach (HAPA; Schwarzer, 1992) posits that health behaviors are influenced by individuals’ intentions, which are in turn shaped by their risk perception, outcome expectancies, and self-efficacy (Renner and Schwarzer, 2005; Schwarzer, 1992). Differences between women and men have been reported for these behavioral antecedents (e.g. for risk perception, Machado Nardi et al., 2020). Hence, gender role orientations might not only explain differences in healthy eating, but also differences in the social-cognitive antecedents of healthy eating, namely risk perception, outcome expectancies, self-efficacy, and the intention to eat healthily.

### Gender role orientations, healthy eating and its social-cognitive antecedents

Risk perception, that is, the awareness of personal health risks, such as the potential risk of cardiovascular diseases due to an unhealthy diet, motivates individuals toward healthier eating behaviors (Renner and Schwarzer, 2005). Women generally exhibit greater risk awareness and sensitivity in health matters than men (Dosman et al., 2001; Weber et al., 2002). This also holds for dietary risk perceptions, where women report greater concerns about nutrition-related risks than men (Machado Nardi et al., 2020; Żarnowski et al., 2022). Those differences between women and men in risk perception may be attributed to distinct gender roles. The feminine gender role is often linked to caretaking and nurturing responsibilities, which makes it also more prone to heightened health and safety concerns (Davidson and Freudenburg, 1996). Contrarily, the masculine gender role often emphasizes risk-taking, risk denial, and invulnerability to disease (Courtenay, 2000a, 2000b). As such, individuals who identify more as compared to less with the feminine gender role might exhibit higher diet-related risk perception, while those who identify more as compared to less with the masculine gender role may perceive these risks as less significant.

Another crucial factor in motivating healthy eating intentions are individuals’ outcome expectancies, that is, their understanding of the consequences of their eating behavior on physical and mental well-being or appearance is important for developing healthy eating intentions (Renner and Schwarzer, 2005). Research indicates that women have a better understanding of the health benefits of a healthy diet and higher outcome expectancies than men (Anderson et al., 2007; Renner and Schwarzer, 2005; Wardle et al., 2004). The close link between the female gender role and engagement with nutrition and food-related activities (Oates and McDonald, 2006; Sullivan, 2000) may also contribute to higher outcome expectancies among individuals who identify more strongly with the feminine gender role compared to those who identify less with this role. With regard to the association between the masculine gender role and outcome expectancies, research is scarce.

Self-efficacy, that is, believing in one’s ability to eat healthily, even under challenging circumstances, is another antecedent of healthy eating (Renner and Schwarzer, 2005). Women exhibit higher self-efficacy regarding healthy eating than men (Anderson et al., 2007; Razaz et al., 2022; Stephens et al., 2017), which may be related to the female gender role’s emphasis on health and nutrition-related behavior (Sobal, 2005). This greater engagement and experience with healthy food choices may provide mastery experiences (Bandura, 1977, 1982) that further strengthen dietary self-efficacy in individuals who strongly identify with the feminine gender role compared to those who identify less with this role. As for outcome expectancies, research regarding the association between the masculine gender role and self-efficacy is scarce.

The intention to eat healthily is the most proximal predictor of healthy eating behavior and is shaped by risk perception, outcome expectancies, and self-efficacy (Renner and Schwarzer, 2005). Women report higher intentions to follow a healthy diet (Gavin et al., 2011; Renner and Schwarzer, 2005) and engage in healthier eating behavior than men (Arganini et al., 2012; Hiza et al., 2013; Imamura et al., 2015). The previously mentioned links between the feminine gender role and risk perception, outcome expectancies, and self-efficacy might translate into a positive relation between the feminine gender role and the intention to eat healthily and healthy eating behavior. In contrast, the characteristics associated with masculinity, such as risk-taking and a disinterest in health and nutrition (Courtenay, 2000b), may hinder the intention to eat healthily and healthy eating behavior. Therefore, individuals who identify more as compared to less with the feminine gender role might show a higher intention to eat healthily and healthier eating behavior. Contrarily those who identify more as compared to less with the masculine gender role might show a lower intention to eat healthily and less healthy eating behavior.

### The present study

In sum, gender role orientations seem a promising avenue for a more nuanced understanding of how gender is related to healthy eating. Although there are studies suggesting that gender role orientations are related to other health behaviors (e.g. Hunt et al., 2007; Sieverding et al., 2005; Zimmermann et al., 2011), no study investigated how they might contribute to the observed differences between women and men in healthy eating and its antecedents according to the Health Action Process Approach (Schwarzer, 1992). To address this gap, the present study investigates whether gender orientations mediate the relation between gender and healthy eating, intention to eat healthily, self-efficacy, outcome expectancies, and risk perception.

We tested the following preregistered hypotheses (see https://doi.org/10.23668/psycharchives.12893):

***H1a*:** *The relationship between gender and risk perception is mediated by femininity scores. Higher femininity scores are associated with increased risk perception.*
***H1b*:** *The relationship between gender and risk perception is mediated by masculinity scores. Higher masculinity scores are associated with decreased risk perception.*
***H2*:** *The relationship between gender and outcome expectancies is mediated by femininity scores. Higher femininity scores are associated with higher outcome expectancies.*
***H3*:** *The relationship between gender and self-efficacy is mediated by femininity scores. Higher femininity scores are associated with higher self-efficacy.*
***H4a*:** *The relationship between gender and intention to eat healthily is mediated by femininity scores. Higher femininity scores are associated with increased intention.*
***H4b*:** *The relationship between gender and intention to eat healthily is mediated by masculinity scores. Higher masculinity scores are associated with decreased intention.*
***H5a*:** *The relationship between gender and healthy eating behavior is mediated by femininity scores. Higher femininity scores are associated with increased healthy eating behavior.*
***H5b*:** *The relationship between gender and healthy eating behavior is mediated by masculinity scores. Higher masculinity scores are associated with decreased healthy eating behavior.*

## Method

### Open practices statement

All hypotheses, sample size, and the data analysis plan were preregistered at ZPID (https://doi.org/10.23668/psycharchives.12893). Raw data and materials are openly available at ZPID (https://doi.org/10.23668/psycharchives.21270).

### Study design and participants

Data were collected in a cross-sectional online survey. A panel provider (Cint) recruited participants with quotas to achieve representativeness for the Austrian adult population concerning gender, age, level of education, and residential area. Participants were invited to participate based on their characteristics (for quotas) and compensated in form of points redeemable for small monetary values. A total of 1025 individuals participated in the study. Participants were excluded if they (1) failed one or both attention checks (*n* = 162; see section Measures) or if they (2) completed the survey in less than half of the median response time (*n* = 38). After applying these exclusion criteria, the final sample consisted of 825 participants. Table 1 presents demographic information of the final sample and Austrian national statistics used for quotas based on the OECD (2022) and Austria’s Federal Statistical Office, Statistik Austria (2020a, 2020b).

**Table 1.** Demographic and anthropometric information of the final sample and Austrian national statistics used for quotas.

Demographic variable	Final sample (*N* = 825)	Austrian national statistics used for quotas^a^
Gender (%)^b^	Women (50.8%) Men (48.7%) Non-binary (0.5%)	Women (50.7%) Men (49.3%) Non-binary (0.003%)
Age (M, SD)^c^	49.1, 16.8	
Age groups (%)	18–29 years old (17.5%) 30–54 years old (41.0%) 55–64 years old (18.3%) 65+ years old (23.3%)	18–29 years old (17.5%) 30–54 years old (42.0%) 55–64 years old (17.5%) 65+ years old (23.0%)
Level of education (%)^d^	Below upper secondary (14,7%) Upper secondary (53,9%) Tertiary (31.0%)	Below upper secondary (14.1%) Upper secondary (51.3%) Tertiary (34.6.0%)
Residential area (%)	Urban (54.4%) Rural (45.6%)	Urban (53.6%) Rural (46.4%)
BMI (M, SD)^c,e^	26.4, 5.2	
BMI category^c,e^	<18.5 (3%) 18.5–<25 (41.3%) 25–<30 (33.3%) 30>(21.7)	<18.5 (2.5%) 18.5–<25 (46.3%) 25–<30 (34.5%) 30> (16.6)

a Age groups according to Statistik Austria (2020a); level of education according to the OECD (2022); residential area according to Statistik Austria (2020b); gender according to Statistik Austria (2025), BMI according to Statistik Austria (2019).

b 50/50 split for men and women was targeted.

c Not used for quotas.

d n.a. = 3.

e n.a. = 5.

### Ethics statement

Before accessing the questionnaire, participants were presented with an online study information sheet outlining the study objectives, confidentiality, voluntary nature of participation, right to withdraw at any time, and research contact details. Participants provided informed consent electronically by actively clicking an agreement checkbox. The study was performed in line with the guidelines of the Declaration of Helsinki. The ethics committee of the Johannes Kepler University approved the study protocol.

### Measures

#### Demographic and anthropometric characteristics

We assessed age, gender (“Which gender do you identify with?”; female/male/I use a different term), and residential area (urban/rural). Level of education was assessed with one question on participants’ highest education level and categorized into below upper secondary, upper secondary, and tertiary education according to the OECD ( 2022). Furthermore, participants indicated their height and weight.

#### Gender role orientations

Gender role orientations were measured by the Gender-Related Attributes Survey (GERAS; for validation details see Gruber et al., 2020), which assesses femininity and masculinity as two separate dimensions along the three areas personality, cognition and activities, and interests. The 25 items of the femininity scale comprise characteristics that have higher social desirability for women than men (e.g. “caring,” “having an interest in shopping”), the 25 items of the masculinity scale comprise characteristics that have higher social desirability for men than women (e.g. “willing to take risks,” “having an interest in weight lifting”). Participants reported for the 50 characteristics the degree to which they match their self-perception on a 7-point Likert scale ranging from 1 (never/not at all) to 7 (always/very much). Scale scores were computed by averaging items. Internal consistencies of the two scales were good (GERAS-Masculinity; Cronbach’s α = 0.86; McDonald’s ω = 0.85; GERAS-Femininity; Cronbach’s α = 0.84; McDonald’s ω = 0.82).

In addition, we administered the German version of the Bem Sex-Role Inventory (BSRI-R; for validation details see Troche and Rammsayer, 2011). Femininity and masculinity were assessed with 15 items each. Scale scores were computed by averaging items. (BSRI-Masculinity; Cronbach’s α = 0.91; McDonald’s ω = 0.91; BSRI-Femininity; Cronbach’s α = 0.89; McDonald’s ω = 0.89). Results for the BSRI-R are reported in Figure 3 in the Appendix.

#### Risk perception

To assess risk perception a validated scale was used (see Gholami and Schwarzer, 2014), specifically by three items introduced by the stem “If I don’t eat a healthy and balanced diet. . .” followed by the statements “. . . then I risk getting cardiovascular diseases,” “. . . then my diet would be harmful to my health,” and “. . . then I might look less attractive” (adapted from Gholami and Schwarzer, 2014). Participants responded on a 4-point Likert scale from 1 (very unlikely) to 4 (very likely), scale scores were computed by averaging items (Cronbach’s α = 0.79; McDonald’s ω = 0.80).

#### Outcome expectancies

Outcome expectancies were assessed with eight items with the stem “If I regularly eat a healthy and balanced diet. . .” followed by statements describing positive consequences like “. . .I feel healthy,” “. . . I’ll feel more comfortable mentally,” or “. . .I am able to control my weight” (adapted from Gholami and Schwarzer, 2014; Keller et al., 2018). Prior research indicated predictive validity of this measure for dietary behavior (Keller et al., 2018). Participants responded on a 4-point Likert scale from 1 (not true at all) to 4 (exactly true), scale scores were computed by averaging items (Cronbach’s α = 0.89; McDonald’s ω = 0.89).

#### Self-efficacy

Self-efficacy was measured by eight items introduced by the stem “I am confident that I can eat a healthy and balanced diet. . .” followed by statements like “. . . even if it is not always easy for me,” “. . . even if it is expensive,” or “. . .even if I cannot see any positive changes immediately” (adapted from Gholami and Schwarzer, 2014; Keller et al., 2018). Prior research indicated predictive validity of this measure for dietary behavior (Keller et al., 2018). Participants indicated their agreement on a 4-point Likert scale from 1 (not true at all) to 4 (exactly true), scale scores were computed by averaging items (Cronbach’s α = 0.82; McDonald’s ω = 0.81).

#### Intention to eat healthily

Intention to eat healthily was measured by the single item “I intend to eat healthy and in a balanced way in the future” on a 7-point Likert scale from 1 (not at all) to 7 (absolutely) (Schwarzer, 2008; Schwarzer and Renner, 2000). This item was shown to be a valid predictor of eating behavior (Klusmann et al., 2019).

#### Healthy eating

Healthy eating was measured by the General Dietary Behavior Inventory (GDBI; for validation details see Engelmann et al., 2021), which captures different components of healthy dietary behavior. The GDBI consists of 16 items using a semantic differential scale to compare dietary behaviors such as “I eat different foods everyday vs. I eat the same foods every day” or “I eat at least 3 servings of vegetables daily vs. I never eat vegetables.” Items were summed to create a total score, participants can score between 16 and 80. Higher scores indicate a healthier dietary behavior.

In addition, participants completed a validated food frequency questionnaire (FFQ; Winkler and Döring, 1995, 1998). Participants indicated how often on average they eat certain food items from 24 food categories (e.g. vegetables, fruits, meat, cake) on a 6-point scale from 1 (almost daily) to 6 (never). A cumulative index according to Winkler and Döring (1995) was calculated, in which reported frequencies were recoded based on their adherence to dietary recommendations. Results for the FFQ are reported in Figure 3 in the Appendix.

#### Attention checks

Two attention checks were incorporated throughout the survey to prevent inattentive responses (e.g. “Please choose ‘not true at all’ at this point”).

### Data analysis and power analysis

With the exception of education and BMI, no missing data occurred, as all other survey items were mandatory. Mediation analyses were conducted using PROCESS macro (Model 4; Hayes, 2022), with age and education entered as covariates because they have consistently been associated with diet quality (e.g. Miller et al., 2022; Olstad and McIntyre, 2025). Bootstrapping with 5000 samples was employed to test the significance of the indirect effect. Effects were considered significant if the 99% confidence intervals did not include zero. Because a total of eight hypotheses were tested, alpha was reduced to 0.006 (Bland and Altman, 1995) and 99% confidence intervals instead of 95% confidence intervals were considered. Because the non-binary subsample was too small for meaningful group analysis (*n* = 4), we restricted the main analyses to participants identifying as women or men.

The required sample size for a mediation model was determined a priori using the Monte Carlo power analysis for indirect effects (Schoemann et al., 2017; rs = 0.14, power = 0.80, α = 0.006; using 10.000 Monte Carlo replications with 20.000 draws), the power analysis indicated that a minimum of 743 participants are required.

## Results

### Descriptive results

Table 2 presents the study variables’ means, standard deviations, and correlations. Table 3 reports descriptive results by gender. Women scored significantly higher than men on femininity, risk perception, outcome expectancies, and the intention to eat healthily. Men scored significantly higher than women on masculinity. There were no significant gender differences regarding self-efficacy and healthy eating.

**Table 2.** Descriptive statistics and correlations between study variables.

Variables	M	SD	1	2	3	4	5	6
1. Masculinity	3.88	0.84						
2. Femininity	4.66	0.73	0.30***					
3. Risk perception	3.15	0.68	0.02	0.19***				
4. Outcome expectancies	3.13	0.57	0.19***	0.35***	0.49***			
5. Self-efficacy	2.70	0.56	0.22***	0.24***	0.19***	0.40***		
6. Intention	5.28	1.42	0.12***	0.32***	0.33***	0.56***	0.47***	
7. Healthy eating	53.72	7.23	0.06	0.21***	0.20***	0.33***	0.32***	0.42***

Note. N = 825.

*** p < 0.001.

**Table 3.** Descriptive results by gender.

	Females (*n* = 419)		Males (*n* = 402)		Gender differences	
Variables	M	SD	M	SD	*t*(819)	Cohen’s *d*
Femininity	4.91	0.67	4.41	0.69	10.54***	0.74
Masculinity	3.63	0.81	4.14	0.79	−9.12***	−0.64
Risk perception	3.21	0.68	3.09	0.67	2.4*	0.17
Outcome expectancies	3.22	0.55	3.04	0.58	4.45***	0.31
Self-efficacy	2.73	0.54	2.66	0.58	1.82	0.13
Intention	5.60	1.27	4.94	1.50	6.79***	0.47
Healthy eating	54.10	7.41	53.32	7.04	1.53	0.11

Note. The table only includes female and male participants; due to the small number of non-binary participants (n = 4), we refrained from a difference test. Gender: 1 = female, 2 = male.

* p < 0.05; ***p < 0.001.

### Can gender role orientations explain gender differences in healthy eating and its antecedents?

**Figure 1.**
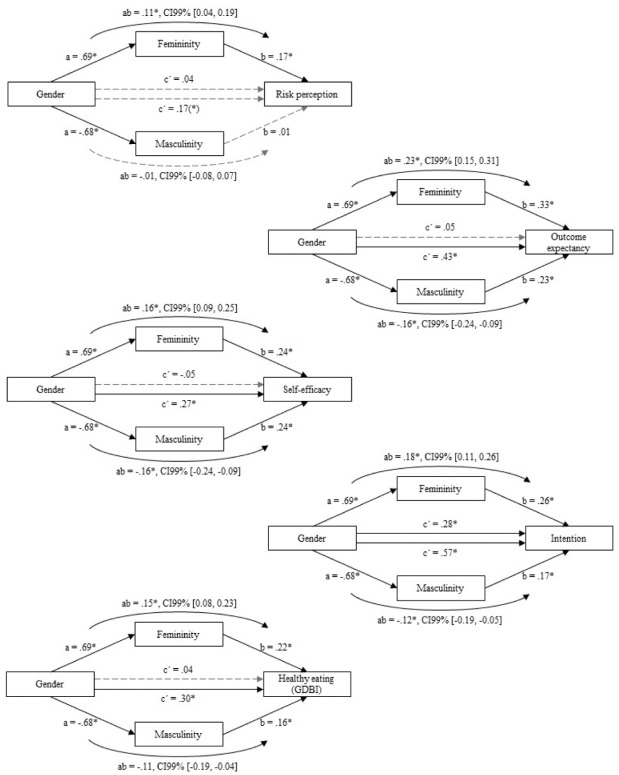
Results of mediation effects of gender through femininity and masculinity on healthy eating and its antecedents. *Note. N* = 818 due to missing values of 3 participants’ level of education and exclusion of 4 non-binary participants. Included covariates: age and education. Coding gender: male and non-binary = 0, female = 1. Path values are standardized coefficients (partially standardized coefficients for dichotomous gender variable). The *ab* path represents the indirect effect of gender on the dependent variables, that is, the effect mediated by femininity or masculinity. The *c’* path refers to the direct effect of gender on the dependent variables (after considering the mediated effect). (*) *p* < 0.05, but due to multiple testing, the significance threshold was reduced to 0.006; *p < 0.001.

The mediation analysis (PROCESS Model 4; Hayes, 2022) indicated that gender indirectly influenced risk perception, outcome expectancies, self-efficacy, the intention to eat healthily, and healthy eating through femininity (see Figure 1 and Table 4 in the Appendix). In line with the hypotheses (**H1a**, **H2**, **H3**, **H4a**, **H5a**), participants with higher femininity scores expressed higher risk perception, outcome expectancies, self-efficacy, intention to eat healthily, and more healthy eating. Hence, the feminine gender role explained the gender difference in healthy eating antecedents.

With regard to the masculine gender role, there was no significant indirect effect of gender on risk perception through masculinity (in contrast to **H1b**). For outcome expectancies, self-efficacy, the intention to eat healthily, and healthy eating there was a significant indirect effect of gender through masculinity (see Figure 1 and Table 4 in the Appendix). However, in contrast to the hypotheses (**H4b**, **H5b**), participants with higher masculinity scores indicated higher outcome expectancies, self-efficacy, intention to eat healthily, and more healthy eating. Hence, although men showed lower outcome expectancies and a lower intention to eat healthily, masculinity was associated with higher outcome expectancies and a higher intention to eat healthily.

Age and education were included as covariates in all models. Education showed consistently positive associations with risk perception, outcome expectancies, self-efficacy, intention to eat healthily and healthy eating; effect sizes were small in absolute magnitude given unstandardized scaling. Age effects were likewise modest and showed mixed associations: it was positively related to healthy eating and negatively to risk perception; for outcome expectancies and self-efficacy, the associations were negative in the femininity models but not significant in the masculinity models. Age was not significantly related to the intention to eat healthily (see Table 4 in the Appendix).

### Exploratory post-hoc item-level correlates of masculinity

**Figure 2.**
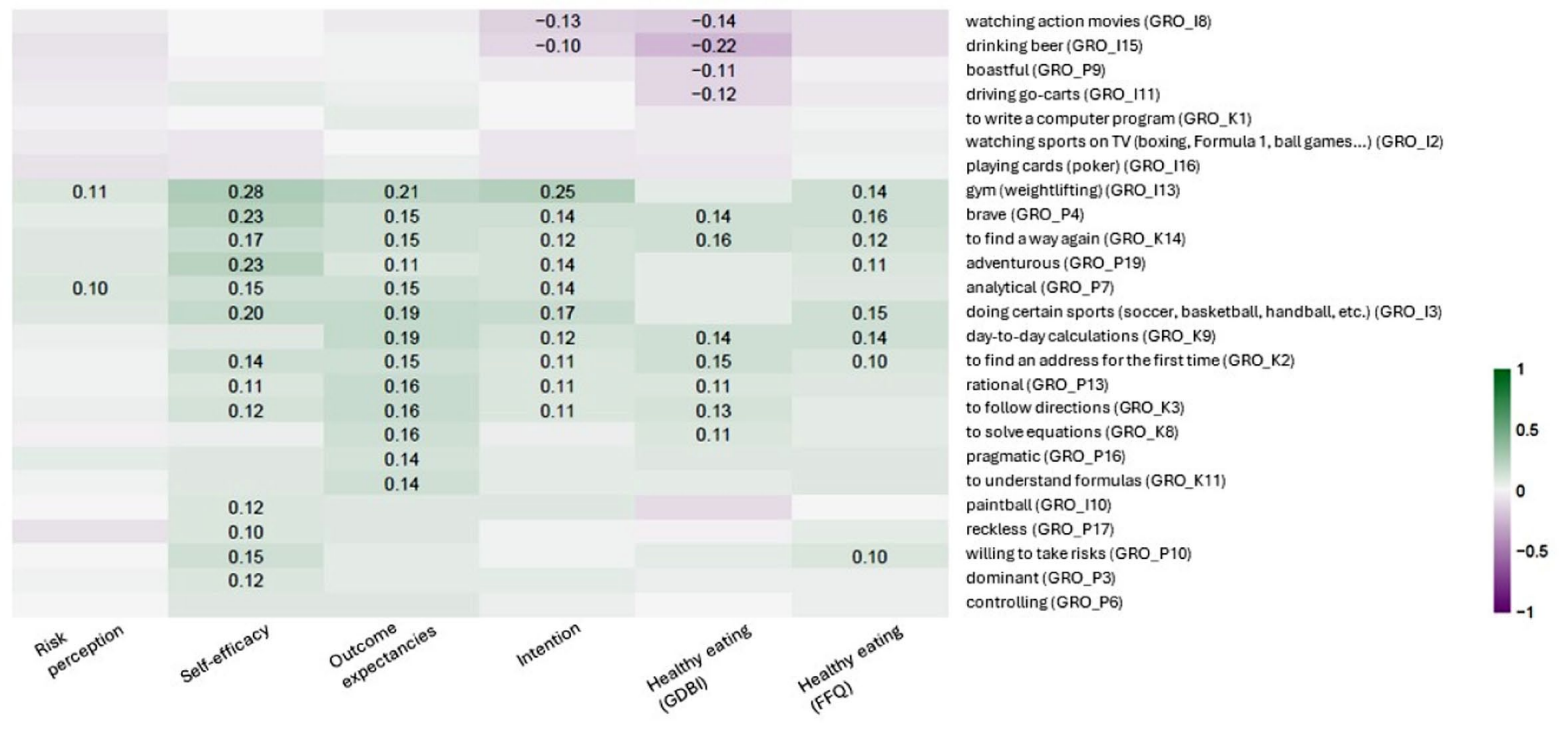
Correlation heatmap of individual masculinity items (GERAS) and healthy eating and its antecedents. *Note*. *N* = 825. Heatmap displays Pearson correlations between individual masculinity items and risk perception, outcome expectancies, self-efficacy, intention to eat healthily and healthy eating. GDBI: General Dietary Behavior Inventory; FFQ: Food Frequency Questionnaire.

To further investigate the unexpected positive associations between masculinity and healthy eating and most of its antecedents observed in the primary analyses, we conducted an exploratory post-hoc item-level analysis. Figure 2 displays pearson correlations between each masculinity item and risk perception, self-efficacy, outcome expectancies, intention to eat healthily and healthy eating (a corresponding heatmap for the femininity items is provided in Appendix Figure 4). Item-level correlations suggest heterogeneous links between masculinity facets and the outcomes. While items such as “weightlifting,” “doing certain sports,” “analytical,” or “rational” showed positive correlations, items such as “drinking beer,” “boastful,” or “watching action movies” showed negative correlations. Given the exploratory, post-hoc nature of this analysis, these patterns should be interpreted cautiously.

## Discussion

The results suggest that gender role orientations can help to understand gender differences in eating behavior and its antecedents. In line with the hypotheses (**H1a**, **H2**, **H3**, **H4a**, **H5a**), femininity positively mediated the relationship between gender and healthy eating, intention to eat healthily, risk perception, outcome expectancies, and self-efficacy. This aligns with prior research linking femininity to health-promoting attitudes and behaviors (e.g. Arganini et al., 2012; Wardle et al., 2004). Interestingly and in contrast to the hypotheses (**H1b**, **H4b**, **H5b**), masculinity was also positively associated with healthy eating and its antecedents, except for risk perception. While this is not in line with previous studies that link masculinity to unhealthy diets and disinterest in nutrition (e.g. Courtenay, 2000b), a potential explanation could be the phenomenon of “trading masculine competence” (de Visser and Smith, 2007), where masculine capital – established through a strong masculine identity in other domains – can act as a buffer against the stigma of engaging in behaviors stereotypically viewed as feminine, like healthy eating (De Visser et al., 2009; de Visser and Smith, 2007). This suggests that individuals with a secure and well-established masculine identity might feel less pressure to conform strictly to traditional masculine norms in all areas of life. Therefore, individuals who demonstrate masculinity in other socially valued ways might have more flexibility to display behaviors that are typically seen as feminine, including healthy eating, without experiencing a threat to their masculinity.

Additionally, femininity and masculinity are not rigid, uniform constructs but rather multifaceted, with the various facets of gender identity potentially interacting differently with particular health behaviors. While both femininity and masculinity were positively associated with healthy eating and most of its antecedents, the underlying motivational structures driving these results might vary. For example, motives stereotypically associated with femininity like caregiving, nurturance, or concerns about physical appearance (e.g. Cronin et al., 2014; Monge-Rojas et al., 2015) can promote health-conscious food choices. The caretaking and nurturing motive might lead individuals to prioritize balanced and nutritious meals for both themselves and their families, perceiving healthy eating as an integral aspect of their caregiving role. Similarly, societal expectations and concerns about physical appearance, which tend to be associated with the feminine gender role, may encourage dietary choices aligned with health promotion. In contrast, masculinity is frequently associated with motives such as optimizing physical strength, and fueling the body for peak functionality (Sloan et al., 2010; Spencer, 2014). In this context, adopting a nutritious diet can serve as a means to enhance performance, sustain endurance, and maintain physical strength. Rather than being perceived as a deviation from traditional masculine norms, healthy eating could be seen as an expression of masculinity when framed within a performance- and strength-oriented mindset. Despite potentially differing motivational pathways, both femininity- and masculinity-related motives might therefore have ultimately led to similar behavioral outcomes. Consistent with this view, our exploratory item-level analysis indicated non-uniform associations across masculinity items. At the same time, the gender role orientation measure we used, captures general characteristics and not directly eating- or health-specific characteristics. Accordingly, links to such motives are interpretive rather than directly observed in our data. Future work should therefore develop and validate domain-specific gender role orientation measures for dietary and health contexts to capture motive-relevant facets more directly. However, observed heterogeneity could indicate that masculinity and healthy eating and its antecedents might be rather multidimensional than unidimensional.

**Figure 3.**
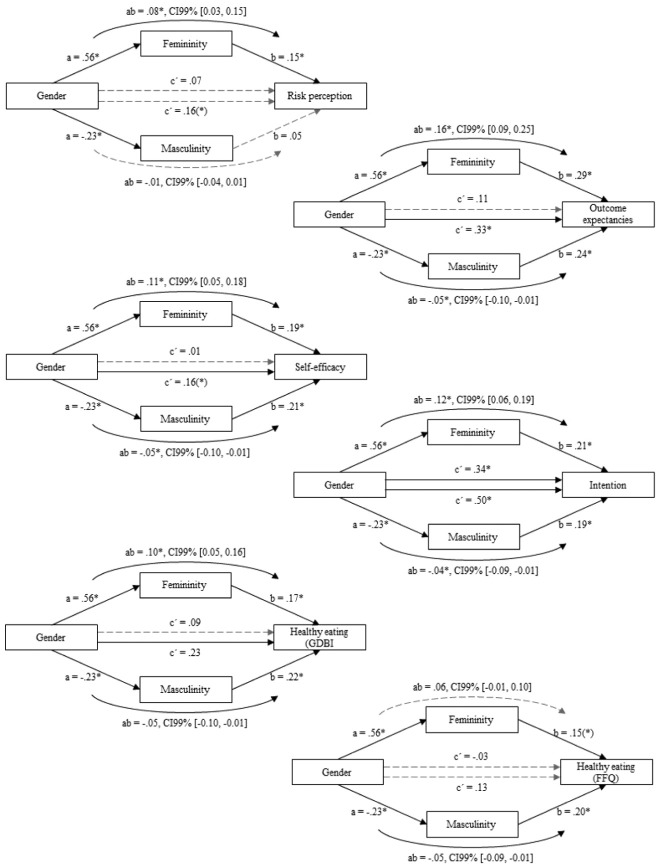
Results of mediation effects of gender through femininity and masculinity (BSRI-R) on healthy eating and its antecedents. *Note. N* = 818 due to missing values of 3 participants’ level of education and exclusion of 4 non-binary participants. Included covariates: age and education. Coding gender: male = 0, female = 1. Path values are standardized coefficients (partially standardized coefficients for dichotomous gender variable). The *ab* path represents the indirect effect of gender on the dependent variables, that is, the effect mediated by femininity or masculinity. The *c’* path refers to the direct effect of gender on the dependent variables (after considering the mediated effect). (*) *p* < 0.05, but due to multiple testing, the significance threshold was reduced to 0.006; *p < 0.001.

Contrary to previous findings (e.g. Arganini et al., 2012; Wardle et al., 2004), there were no gender differences in healthy eating and self-efficacy. One explanation could be that the binary conceptualization of gender is increasingly insufficient and that taking into account more nuanced measures like gender role orientations can provide more insights into gendered aspects of health behavior. Furthermore, the absence of gender differences in self-efficacy might reflect broader sociocultural shifts in health-related attitudes. As nutrition becomes an increasingly accessible topic, and as both women and men gain more similar exposure to nutrition-related information and opportunities for healthy food choices, their self-efficacy levels may increasingly converge. Another consideration is that previous studies often focused on selected aspects of healthy eating (e.g. fruit and vegetable consumption or restrained eating; Legget et al., 2023; Prättälä et al., 2007), which do not fully capture the multidimensional nature of healthy eating behavior. The General Dietary Behavior Inventory (GDBI; Engelmann et al., 2021) used in this study assesses a broader range of eating facets rather than isolated food groups. This might explain why no gender differences emerged – while some aspects of a healthy diet are more likely to be adopted by women, others are more likely to be adopted by men, and additional non-gendered aspects might further offset gender differences in healthy eating. Importantly, similar patterns were also found with the Food Frequency Questionnaire (FFQ; Winkler and Döring, 1995), which likewise indicated a positive association of both femininity and masculinity with healthy eating (see Figure 3 in the Appendix).

### Strengths and limitations

With a large and representative sample of the Austrian population, this study provides robust insights into gendered eating behavior within this cultural context. Moreover, the associations between gender role orientations and healthy eating and its antecedents were controlled for age and education. Because the sampling approximated the Austrian adult population with regard to age, education, and residential area, the results are generalizable across those characteristics. Additionally, the study’s focus on gender role orientations rather than gender categories allows for a more differentiated understanding of gendered eating behavior, offering new insights beyond traditional gender comparisons. However, there are also limitations to mention. First, eating behavior (Hopwood et al., 2024; Sproesser et al., 2019) and conceptions of femininity and masculinity can vary across cultures (Watkins et al., 1998). The results from an Austrian sample therefore are certainly limited in scope. For example, a cross-cultural comparison involving 23 countries indicated that the level of gender differences in meat consumption varied depending on the level of gender equality in the respective countries (Hopwood et al., 2024). Cultural values and norms might, thus, affect how healthy eating behavior is linked to feminine and masculine identities. Second, the study used self-reported measures of eating behavior, which might be subject to social desirability bias or inaccuracies in recall. Third, while the study identifies associations between gender role orientations and healthy eating and its antecedents, the cross-sectional design does not allow causal inferences. Longitudinal or experimental designs (e.g. Renner et al., 2012, 2016) are needed to establish the directionality of these relationships. Fourth, as the study focused on the role of gender role orientations, we did not extend the models to include the HAPA constructs as additional mediators. Finally, we only included age and education as covariates, although other variables such as chronic health conditions or responsibility for foodwork may also shape healthy eating and its antecedents.

### Implications for future research

This study was a first step toward a more differentiated perspective on the relation of gender and healthy eating and its antecedents according to the HAPA framework. More research – also beyond cross-sectional studies and self-reported eating behavior– is needed to fully understand the complex nature of gendered eating behavior.

The positive association of masculinity with healthy eating and most of its antecedents raises further questions. Future research should explore under which conditions masculinity can serve as a health-promoting vehicle and how this can be actively harnessed to support healthy eating behavior and design gender-sensitive interventions. Disentangling different dimensions of masculinity, such as strength, willingness to take risks, or control, could help clarify which aspects of masculinity can contribute to healthy food choices.

The findings also highlight the need for gender-sensitive health interventions that move beyond binary gender categorizations, as understanding the potentially differently gendered motivational pathways to healthy eating better could yield critical implications for optimal health interventions. Lastly, addressing the cultural and contextual contingencies that affect the relationships between the concepts of femininity and masculinity and health behaviors, including eating behavior, would provide a basis for more effective and inclusive health promotion.

## Figures and Tables

**Figure 4.**
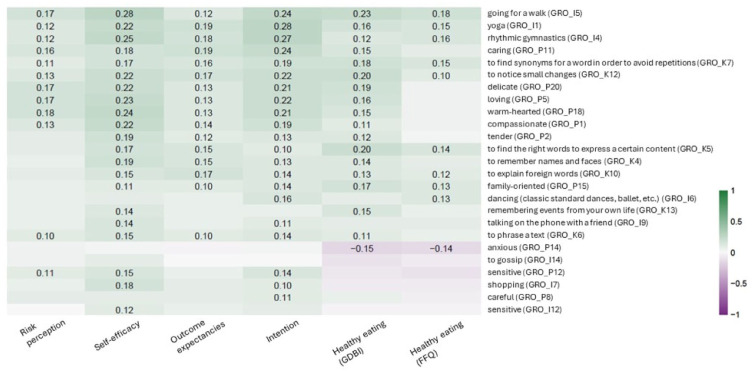
Correlation heatmap of individual femininity items (GERAS) and healthy eating and its antecedents. *Note. N* = 825. Heatmap displays Pearson correlations between individual masculinity items and risk perception, outcome expectancies, self-efficacy, intention to eat healthily and healthy eating. GDBI: General Dietary Behavior Inventory; FFQ: Food Frequency Questionnaire.

## Appendix

**Table 4.** Results of mediation effects of gender through femininity and masculinity.

Model 1: gender → femininity → risk perception								
		Consequent						
		M (femininity)				Y (risk perception)		
Antecedent		Coeff.	SE	*p*		Coeff	SE	*p*
X (gender)	*a*	0.498	0.048	<0.001	*c‘*	0.024	0.049	0.634
M (femininity)		-	-	-	*b*	0.155	0.034	<0.001
Education		0.104	0.036	0.004		0.072	0.036	0.044
Age		−0.002	0.001	0.241		−0.003	0.001	0.029
Constant		4.264	0.119	<0.001		2.412	0.187	<0.001
		*R*^2^ = 0.131 *F*(3, 814) = 41.864, *p* < 0.001				*R*^2^ = 0.047 *F*(4, 813) = 10.035, *p* < 0.001		
		Indirect effect						
		Effect		SE			[99% CI]	
	*ab*	0.077		0.020			[0.027, 0.133]	
Model 2: gender → masculinity → risk perception								
		Consequent						
		M (masculinity)				Y (risk perception)		
Antecedent		Coeff.	SE	*p*		Coeff.	SE	*p*
X (gender)	*a*	−0.575	0.053	<0.001	*c‘*	0.107	0.051	0.036
M (masculinity)		-	-	-	*b*	0.010	0.032	0.75
Education		0.173	0.04	<0.001		0.086	0.036	0.018
Age		−0.016	0.002	<0.001		−0.003	0.002	0.036
Constant		4.57	0.132	<0.001		3.026	0.186	<0.001
		*R*^2^ = 0.217 *F*(3, 814) = 75.287, *p* < 0.001				*R*^2^ = 0.028 *F*(4, 813) = 4.752, *p* < 0.001		
		Indirect effect						
		Effect		SE			[99% CI]	
	*ab*	−0.006		0.019			[−0.054, 0.048]	
Model 3: gender → femininity → outcome expectancies								
		Consequent						
		M (femininity)				Y (outcome expectancies)		
Antecedent		Coeff.	SE	*p*		Coeff.	SE	*p*
X (gender)	*a*	0.498	0.048	<0.001	*c‘*	0.028	0.040	0.478
M (femininity)		-	-	-	*b*	0.260	0.027	<0.001
Education		0.149	0.036	0.004		0.056	0.028	0.051
Age		−0.002	0.001	0.241		−0.004	0.001	<0.001
Constant		4.264	0.119	<0.001		1.993	0.149	<0.001
		*R*^2^ = 0.131 *F*(3, 814) = 41.024, *p* < 0.001				*R*^2^ = 0.146 *F*(4, 813) = 34.863, *p* < 0.001		
		Indirect effect						
		Effect		SE			[99% CI]	
	*ab*	0.225		0.033			[0.145, 0.313]	
Model 4: gender → masculinity → outcome expectancies								
		Consequent						
		M (masculinity)				Y (outcome expectancies)		
Antecedent		Coeff.	SE	*p*		Coeff.	SE	*p*
X (gender)	*a*	−0.575	0.053	<0.001	*c‘*	0.248	0.041	<0.001
M (masculinity)		-	-	-	*b*	0.159	0.026	<0.001
Education		0.173	0.040	<0.001		0.055	0.029	0.062
Age		−0.016	0.002	<0.001		−0.002	0.001	0.105
Constant		4.578	0.132	<0.001		2.367	0.151	<0.001
		*R*^2^ = 0.217 *F*(3, 814) = 75.287, *p* < 0.001				*R*^2^ = 0.096 *F*(4, 813) = 21.487, *p* < 0.001		
		Indirect effect						
		Effect		SE			[99% CI]	
	*ab*	−0.160		0.028			[−0.237, −0.088]	
Model 5: gender → femininity → self-efficacy								
		Consequent						
		M (femininity)				Y (self-efficacy)		
Antecedent		Coeff.	SE	*p*		Coeff	SE	*p*
X (gender)	*a*	0.498	0.048	<0.001	*c‘*	−0.027	0.040	0.494
M (femininity)		-	-	-	*b*	0.181	0.028	<0.001
Education		0.104	0.036	0.004		0.128	0.029	<0.001
Age		−0.002	0.001	0.241		−0.002	0.001	0.039
Constant		4.264	0.119	<0.001		1.702	0.151	<0.001
		*R*^2^ = 0.131 *F*(3, 814) = 41.024, *p* < 0.001				*R*^2^ = 0.091 *F*(4, 813) = 20.387, *p* < 0.001		
		Indirect effect						
		Effect		SE			[99% CI]	
	*ab*	0.162		0.031			[0.089, 0.250]	
Model 6: gender → masculinity → self-efficacy								
		Consequent						
		M (masculinity)				Y (self-efficacy)		
Antecedent		Coeff.	SE	*p*		Coeff.	SE	*p*
X (gender)	*a*	−0.575	0.053	<0.001	*c‘*	0.154	0.041	<0.001
M (masculinity)		-	-	-	*b*	0.158	0.025	<0.001
Education		0.173	0.040	<0.001		0.119	0.029	<0.001
Age		−0.016	0.002	<0.001		−0.000	0.002	0.913
Constant		4.578	0.132	<0.001		1.749	0.149	<0.001
		*R*^2^ = 0.217 *F*(3, 814) = 75.287, *p* < 0.001				*R*^2^ = 0.087 *F*(4, 813) = 19.479, *p* < 0.001		
		Indirect effect						
		Effect		SE			[99% CI]	
	*ab*	−0.163		0.029			[−0.241, −0.089]	
Model 7: gender → femininity → intention								
		Consequent						
		M (femininity)				Y (intention)		
Antecedent		Coeff.	SE	*p*		Coeff	SE	*p*
X (gender)	*a*	0.498	0.048	<0.001	*c‘*	0.391	0.099	<0.001
M (femininity)		-	-	-	*b*	0.501	0.068	<0.001
Education		0.104	0.036	0.004		0.342	0.071	<0.001
Age		−0.002	0.001	0.241		−0.005	0.003	0.097
Constant		4.264	0.119	<0.001		2.186	0.371	<0.001
		*R*^2^ = 0.133 *F*(3, 814) = 41.024, *p* < 0.001				*R*^2^ = 0.153 *F*(4, 813) = 36.610, *p* < 0.001		
		Indirect effect						
		Effect		SE			[99% CI]	
	*ab*	0.179		0.031			[0.163, 0.263]	
Model 8: gender → masculinity → intention								
		Consequent						
		M (masculinity)				Y (intention)		
Antecedent		Coeff.	SE	*p*		Coeff.	SE	*p*
X (gender)	*a*	−0.575	0.053	<0.001	*c‘*	0.816	0.101	<0.001
M (masculinity)		-	-	-	*b*	0.296	0.063	<0.001
Education		0.173	0.040	<0.001		0.345	0.073	<0.001
Age		−0.016	0.002	<0.001		−0.001	0.003	0.792
Constant		4.578	0.120	<0.001		3.001	0.373	<0.001
		*R*^2^ = 0.217 *F*(3, 814) = 75.287, *p* < 0.001				*R*^2^ = 0.117 *F*(4, 813) = 27.122, *p* < 0.001		
		Indirect effect						
		Effect		SE			[99% CI]	
	*ab*	−0.166		0.039			[−0.273, −0.068]	
Model 9: gender → femininity → healthy eating (GDBI)								
		Consequent						
		M (femininity)				Y (healthy eating)		
Antecedent		Coeff.	SE	*p*		Coeff	SE	p
X (gender)	*a*	0.498	0.048	<0.001	*c‘*	0.257	0.514	0.617
M (femininity)		-	-	-	*b*	2.163	0.353	<0.001
Education		0.104	0.036	0.004		1.450	0.367	<0.001
Age		−0.002	0.001	0.241		0.109	0.015	<0.001
Constant		4.264	0.119	<0.001		34.973	1.913	<0.001
		*R*^2^ = 0.131 *F*(3, 814) = 41.024, *p* < 0.001				*R*^2^ = 0.117 *F*(4, 813) = 26.964, *p* < 0.001		
		Indirect effect						
		Effect		SE			[99% CI]	
	*ab*	1.079		0.208			[0.584, 1.668]	
Model 10: gender → masculinity → healthy eating (GDBI)								
		Consequent						
		M (masculinity)				Y (healthy eating)		
Antecedent		Coeff.	SE	*p*		Coeff.	SE	*p*
X (gender)	*a*	−0.575	0.053	<0.001	*c‘*	2.144	0.522	<0.001
M (masculinity)		-	-	---	*b*	1.405	0.325	<0.001
Education		0.173	0.040	<0.001		1.433	0.375	<0.001
Age		−0.016	0.002	<0.001		0.128	0.015	<0.001
Constant		4.578	0.132	<0.001		37.764	1.921	<0.001
		*R*^2^ = 0.217 *F*(3, 814) = 75.287, *p* < 0.001				*R*^2^ = 0.097 *F*(4, 813) = 47.640, *p* < 0.001		
		Indirect effect						
		Effect		SE			[99% CI]	
	*ab*	−0.112		0.292			[−1.192, −0.038]	

*Note. N* = 818, due to missing values of 3 participants’ level of education and exclusion of 4 non-binary participants. Coding gender: male = 0, female = 1. Coefficients and standard errors (SE) are reported in unstandardized form.

## Notes

1. In this study, we focus on gender as a social and psychological construct rather than biological sex. Whereas sex refers to a multidimensional construct based on physiological and anatomical traits and typically assigned at birth based on visible markers, gender describes the social and psychological dimensions of identity, including how individuals perceive themselves and how they are perceived by others in relation to cultural norms and expectations (National Academies of Sciences, Engineering, and Medicine, 2022).
